# Examining the relationship between peer support and foreign language emotions in online learning: the mediating effect of self-efficacy

**DOI:** 10.3389/fpsyg.2023.1148472

**Published:** 2023-09-29

**Authors:** Yujie Huang

**Affiliations:** Department of Foreign Languages, Wuhan Polytechnic University, Wuhan, China

**Keywords:** peer support, foreign language emotions, enjoyment, anxiety, self-efficacy, online learning

## Abstract

**Introduction:**

With the proliferation of online learning, understanding students’ affective experiences in digital foreign language courses has become imperative.

**Methods:**

This mixed-methods study investigates how peer support and self-efficacy shape the emotional experiences of 502 Chinese undergraduate students in online English courses. Quantitative analyses using surveys were conducted to examine the relationships between peer support, self-efficacy, enjoyment, and anxiety. Qualitative analyses of interviews provided additional insights.

**Results:**

Quantitative analyses reveal that peer support positively predicts higher levels of foreign language enjoyment and anxiety. Self-efficacy was found to partially mediate this relationship, with peer support strengthening self-efficacy, which, in turn, positively influences enjoyment and anxiety. Qualitative analyses provide converging yet nuanced insights into how peer support enhances self-efficacy and emotions beyond the surveys.

**Discussion:**

The findings provide insight into optimizing online language courses through peer support and self-efficacy development tailored to diverse student needs. Limitations around sample selection, self-reported measures, and scope of relationships examined present opportunities for future research.

## Introduction

The widespread adoption of technology and e-learning platforms has revolutionized the educational landscape, particularly in the context of the COVID-19 pandemic. Such platforms offer flexibility and convenience and introduce new dynamics in students’ emotional experiences. Understanding emotions in learning, particularly in second language learning, is a well-established psychological and educational research area. Emotions are complex psychological states involving three distinct components: a subjective experience, a physiological response, and a behavioral or expressive response (Izard, 2009). In language learning, emotions have been found to significantly influence cognitive processes, maintain interest in learning materials, impact information processing modes, and affect engagement and self-regulation of learning (Dewaele et al., 2019).

Previous research has highlighted the importance of social support in enhancing students’ academic performance, emotions, and self-efficacy (Bandura, 2006; Altermatt, 2019). However, the relationship between peer support, self-efficacy, and emotions in online language learning remains unclear and requires further investigation.

While some studies have shown that peer support can enhance self-regulation and self-efficacy in online language learning (Peeters and Mynard, 2021), the mechanisms underlying this relationship are poorly understood. Thus, it is essential to investigate the potential mediating role of self-efficacy in the relationship between peer support and emotions in online English classes.

Recent findings support that self-efficacy can mediate the relationship between emotions and academic performance (Oriol-Granado et al., 2017). Given the prevalence of enjoyment and anxiety in academic emotions (Pekrun et al., 2009), this study aims to extend this line of inquiry into online English classes. Specifically, this study will investigate the mediating role of self-efficacy between enjoyment, anxiety, and peer support in this context.

This study is of critical importance in the current educational landscape, as the findings will inform the development of more effective online courses that promote positive academic emotions, enhance learning outcomes, and provide educators with a better understanding of the interplay between these constructs in an online learning environment. Furthermore, the research will contribute to a deeper understanding of the emotional experiences of language learners in online settings, and how they can be supported to achieve better educational outcomes.

### Peer support and foreign language emotions

A growing body of research has examined connections between peer support and emotional experiences in foreign language learning (Al-Amri, 2020). Peer interactions are integral in influencing learner affect and motivation. This relationship has been studied across diverse languages, educational contexts, and instructional modalities (Shao and Kang, 2022). A valuable framework for conceptualizing the link between peer support and emotions is the control-value theory of achievement emotions proposed by Pekrun (2006). This theory posits that learners’ emotions are shaped by their subjective control appraisals and value appraisals regarding achievement activities. Peer interactions likely influence students’ perceptions of control and task value, consequently impacting learning-related emotions.

The control-value theory proposes that students experience positive activating emotions like enjoyment when they feel in control of achievement and value those activities (Shao et al., 2023). In contrast, lack of control and value elicits negative deactivating emotions like boredom (Li, 2021). Excessive demands result in negative activating emotions like anxiety, while meager demands produce relaxation (Shao et al., 2023). Research indicates peer support enhances students’ perceived control over language learning tasks, contributing to positive emotions. Collaborative peer interactions and scaffolding assistance foster feelings of competence. Peers also influence subjective task values, increasing perceptions that language learning is engaging, meaningful, and valuable (Tze et al., 2022).

For instance, a study by Shao et al. (2023) examined the influence of students’ perceived online control-value appraisals on their emotions in an online EFL learning context during the COVID pandemic. The study found that control-value appraisals significantly affected students’ emotions, including enjoyment, hope, pride, anxiety, and shame (Shao et al., 2023). Another study by Tze et al. (2022) found that peer support enhanced students’ perceived control over language learning tasks, contributing to positive emotions. The study also revealed that collaborative peer interactions and scaffolding assistance fostered feelings of competence. In contrast, peers influenced subjective task values, increasing perceptions that language learning is engaging, meaningful, and valuable (Tze et al., 2022).

In online language classes, peer support can help mitigate negative emotions by optimizing control-value appraisals (Spagnolo et al., 2019). Affective factors in distance learning programs were examined, and surveys and interviews indicated that regular peer interactions decreased anxiety and alienation. Learners emphasized the value of classmates’ emotional support during strenuous activities, and perceived peer availability enhanced control perceptions by providing help when needed (Spagnolo et al., 2019). For students in an online EFL course, peer connectivity minimized isolation and anxiety. However, some students struggled to make connections, exacerbating negative emotions (Waite, 2021). This underscores the importance of facilitating meaningful peer support in digital environments.

Moreover, recent research suggests that casual social media interactions can impact language learners’ emotions beyond formal language classes by shaping their control-value appraisals (Lu and Wang, 2023). Emotions have a social component and can be influenced by social situations. For instance, face-to-face conversations can elicit socially structured emotional reactions that are not immediately apparent to the individual (Little, 2018).

Similarly, social interactions can shape learners’ control-value appraisals and influence their emotions toward language learning (Shao et al., 2020). However, negative social interactions, such as limited peer engagement, can harm learners’ emotional experiences and language learning outcomes (Alamer and Almulhim, 2021).

While research has shown a link between peer support and positive learner affect, further investigation is needed to understand this relationship fully. Studies have primarily focused on learner perceptions at specific time points, lacking research on the sustainability of social support effects over time. There needs to be more detailed research on how online peer support impacts students’ sense of control and task value differently.

Existing findings emphasize the importance of peer support for developing adaptive motivational beliefs and positive emotions in students, regardless of the learning context. However, empirical findings need to be explicitly connected to the control-value theory to strengthen this argument. Doing so would provide a more comprehensive understanding of this complex phenomenon. Ultimately, uncovering the causal mechanisms underlying peer support is critical to optimizing peer learning implementations and facilitating learner persistence.

Future research should tackle the complex mechanisms through which peer support influences motivation and emotions, utilizing control-value theory as a guiding framework. Studies are needed to determine the sustainability of social support effects over time and the impact of online peer support on students’ sense of control and task value. A pathway to a complete understanding of peer support’s causal mechanisms should be mapped out from empirical data connected explicitly to control-value theory, ultimately laying the foundation to optimize peer learning interventions and inform educational practice. Addressing these gaps would provide a more comprehensive picture of how peer support functions to enhance language learning.

### The role of self-efficacy in foreign language emotions: enjoyment and anxiety

Research has revealed that self-efficacy is pivotal in shaping students’ emotional experiences in foreign language education. In particular, self-efficacy has been shown to strongly influence two critical emotions in language learning: enjoyment and anxiety (Piniel and Csizér, 2013).

Enjoyment involves positive emotions like pleasure and excitement during language learning. Studies show self-efficacy strongly predicts enjoyment, as students with higher self-efficacy enjoy using the language more in class. Their positive appraisals and proactive engagement may enhance enjoyment. By fostering enjoyable experiences, self-efficacy promotes engagement and achievement (Graham, 2022).

In contrast, anxiety stems from nervousness and worry about language learning and use. Numerous studies reveal an inverse relationship between self-efficacy and anxiety across diverse contexts. As social cognitive theory suggests, higher self-efficacy reduces anxiety by enabling adaptive appraisals and expectations of success (Bandura, 1997; Mills et al., 2006).

However, some studies reveal conditional relationships between self-efficacy and anxiety dependent on individual learner differences. This indicates that the connection may be more complex than a straightforward negative correlation. Additional research is required to understand the nuanced interplay between self-efficacy and anxiety fully (Paul and Glassman, 2017). Additionally, a few studies reveal positive correlations, suggesting heightened self-efficacy could elevate anxiety by increasing pressures to meet high-performance standards (Barrows et al., 2013).

Moreover, self-efficacy and anxiety likely influence each other reciprocally over time. While higher self-efficacy generally associates with lower anxiety and greater enjoyment, decreases in self-efficacy may temporarily provoke anxiety. Longitudinal mixed methods studies are needed to elucidate this dynamic relationship.

Substantial evidence shows self-efficacy is pivotal in shaping critical foreign language emotions – enjoyment and anxiety. However, this relationship’s complex mechanisms warrant further investigation across diverse contexts and individuals. Fully understanding these nuances will inform efforts to optimize students’ emotional experiences.

### Sources and development of self-efficacy in foreign language emotions

The pivotal role of self-efficacy in foreign language emotions has been recognized in academic research, particularly emphasizing the necessity of nurturing adaptive self-efficacy beliefs among learners. Rooted in social cognitive theory, self-efficacy is a comprehensive construct primarily shaped by four key sources: mastery experiences, vicarious experiences, verbal persuasion, and physiological states (Bandura, 1997).

Mastery experiences, defined as successful task performances, are considered the most potent source of self-efficacy (Yokoyama, 2019). For instance, a learner scoring highly on an online quiz might cultivate an enhanced belief in their capacity to understand the study materials (Yokoyama, 2019).

Vicarious experiences, or observing peers successfully executing tasks, can also bolster self-efficacy. This form of social modeling can alleviate anxiety associated with impending challenges (Harrison and McGuire, 2006).

Verbal persuasion, encompassing affirmations and laudations from educators and classmates, can further reinforce students’ confidence in their linguistic competence (Cadiz-Gabejan, 2021).

Lastly, physiological states can influence self-efficacy perceptions. A learner who experiences anxiety during an online exam can reframe their physiological responses as a sign of excitement rather than trepidation, thereby fostering adaptive self-efficacy beliefs (Aikens and Kulacki, 2023).

Understanding the developmental pathways of these self-efficacy sources in digital contexts can provide valuable insights into constructing online language learning environments that promote adaptive motivational evaluations and positive emotional responses. Specifically, online peer support can significantly influence the formation of self-efficacy beliefs by facilitating mastery experiences, demonstrating successful modeling, and offering encouragement.

Self-efficacy, which emerges from various sources, is integral to foreign language emotions. Enhancing adaptive self-efficacy through online peer interactions can provide emotional support to learners. However, a more comprehensive understanding of these intricate dynamics requires further investigation.

### The impact of peer support on the development of self-efficacy in online learning

While peer support shows promise in fostering self-efficacy in digital learning environments, its underlying mechanisms still need to be fully understood. Therefore, optimizing online peer support initiatives to enhance self-efficacy is crucial for empowering students to succeed in digital contexts.

Collaborating with peers on academic tasks can enable mastery experiences that boost self-efficacy among online learners. Peer tutoring provides scaffolded mastery experiences while co-creating videos on course topics strengthens self-belief in capabilities. Observing peers succeed at academic challenges can raise observers’ beliefs in their capabilities through social modeling while reducing anxiety about impending tasks. Discussions, where students share study tactics and strategies for overcoming setbacks, can provide vicarious self-efficacy boosts, conveying that challenges are surmountable through effort and peer support (Han and Geng, 2023). Additionally, online peer feedback and encouragement can promote self-efficacy, providing motivational boosts and modeling growth mindsets (Zhang et al., 2020).

Shared struggles create online communities, helping combat isolation and normalizing difficulties, facilitating adaptive self-efficacy perceptions. To design effective online peer support initiatives that promote self-efficacy and enhance student success in digital contexts, it is crucial to understand the mechanisms through which online peer support develop empowering self-efficacy.

### The mediating effect of self-efficacy on the relationship between peer support and foreign emotions in online learning

With the proliferation of online and blended models, foreign language education is transforming the digital landscape. This transformation has led to a need to understand how socio-emotional factors impact learners’ experiences within these technologically-mediated milieus. Specifically, the interdependence between peer support, self-efficacy, and foreign language emotions is particularly interesting.

Although peer support and self-efficacy have been individually linked to foreign language emotions, recent research suggests that self-efficacy may be an intermediary between peer support and affect in computer-assisted language learning (Dong et al., 2022; Zhang, 2022). Investigating this hypothesized mediation can provide valuable insights into how different forms of peer support shape students’ affective encounters in online settings.

Existing literature indicates positive associations between self-efficacy and pleasant emotions like enjoyment, coupled with negative correlations or uncertainty between self-efficacy and unpleasant emotions like anxiety in online foreign language education (Wang and Li, 2022). Peer support can influence students’ self-assessments of language competence, which in turn informs their emotional experiences.

Recent studies provide preliminary empirical evidence for self-efficacy mediating between peer support and foreign language emotions in online contexts (Zhao and Liu, 2022; Derakhshan and Fathi, 2023). However, current research has limitations, including self-report scales, a lack of differentiation between emotional, informational, and appraisal support types, and a focus on traditional classrooms rather than computer-mediated peer interactions.

To address these limitations, future research should integrate qualitative techniques like interviews and observations with quantitative scales to understand better how different types of peer support shape self-efficacy beliefs and consequent foreign language emotions in online settings.

Based on preliminary evidence for the mediating role of self-efficacy, this study proposes a model in which self-efficacy beliefs mediate the impact of peer support on emotions in online foreign language classes. The study aims to test this proposed mediation model by addressing the following research questions:

RQ1: how do different forms of peer support (emotional, informational, and evaluative) influence students’ self-efficacy in online foreign language classes?

RQ2: what is the relationship between self-efficacy shaped by peer support and key foreign language emotions (enjoyment and anxiety), considering gender differences?

RQ3: what classroom contextual factors moderate the relationships between peer support, self-efficacy, and emotions?

The current study seeks to provide practical insights for designing online foreign language courses by elucidating the mediating mechanism by which peer support enhances self-efficacy and optimizes learning emotions. This is critical for supporting online language learners’ social and emotional needs and motivation, who may face unique challenges such as isolation and lack of face-to-face interaction.

The study will examine the variables of peer support, self-efficacy, and learning emotions and their interrelationships. Mixed methods research incorporating quantitative and qualitative approaches can supplement and expand on these preliminary findings, allowing for a more comprehensive understanding of the relationship between peer support, self-efficacy, and learning emotions in online language learning.

## Methodology

### Research context

Chinese institutions offered the Online College English Reading and Writing Course III to improve students’ English reading, writing, critical thinking, and cross-cultural communication skills by exploring current topics. The course utilized various approaches, including vocabulary building, text structure analysis, language skills, and cultural knowledge. Additionally, the course incorporated video lectures and live conferencing to enhance the learning experience.

### Participants and procedures

The participants were recruited from undergraduate students who registered for the online English reading and writing course at two Chinese universities in Wuhan. In this research, 502 participants were invited to complete an online questionnaire (Wenjuanxing, the equivalent of SurveyMonkey in China). Data collection was administered two weeks before the end of the term. After removing those with incomplete responses, 481 (221 males, 260 females) questionnaires remained, yielding a 95.82% valid response rate. The specific distribution of the study is shown in Table 1. Ten students (four males and six females) participated in the subsequent interview.

**Table 1:** Information of interviewees.

	Category	Number	Percentage (%)
Gender	Male	221	45.95
	Female	260	54.05
Nationality	Han	433	90.02
	Minority	48	9.98
Years of learning English	1–6	52	10.81
	7–10	211	43.87
	10–13	218	45.32
Satisfaction with the class	Dissatisfied	6	1.25
	Ok	125	25.99
	Very satisfied	350	72.77

### Measures

#### Quantitative measures

a. Foreign Language Enjoyment Scale (FLE).

The Chinese version of the FLE (Li et al., 2018) was used to assess students’ enjoyment of English class. It consists of 11 items on a five-point Likert scale. Internal consistency was high (Cronbach’s alpha = 0.91).

b. Foreign Language Classroom Anxiety Scale (FLCA).

The FLCA (Horwitz et al., 1986) contains 8 items on a five-point Likert scale. Internal consistency was high (Cronbach’s alpha = 0.91).

c. General Self-Efficacy Scale (GSE).

The GSE (Wang, 2001) comprises 10 items on a four-point Likert scale. Internal consistency was satisfactory (Cronbach’s alpha = 0.94).

d. Peer Support Scale (PS).

The PS (Johnson et al., 1983) contains 9 items assessing perceived peer support. A five-point Likert scale was used. Internal consistency was high (Cronbach’s alpha = 0.9).

e. Semi-structured interviews.

Semi-structured interviews were conducted to gain in-depth insights into students’ perspectives relevant to the research questions.

The main questions focused on four areas: (1) Peer support’s influence on self-efficacy, for example, “How do your peers provide support in your online English class? (prompt for emotional, informational, and evaluative support examples) How does collaborating with your peers influence your confidence in using English? Can you provide any examples?” (2) Relationship between self-efficacy and emotions, for example, “When you feel confident in your English abilities, how does that impact your enjoyment of learning activities? Please explain.” (3) Contextual factors moderating peer support, for example, “How do aspects of the online class environment, like class size or teacher interaction, influence the support you get from peers?” (4) Overall impact of peers on confidence, enjoyment, and anxiety, for example, “If you could advise instructors on facilitating peer support online, what would it be?” The open-ended interviews allowed a more comprehensive understanding of students’ experiences.

### Data analysis

The quantitative data underwent screening for missing values, outliers, and reliability in addition to descriptive statistics and normality tests. SPSS 26.0 was used for data analyses, including multiple regression analyses to test the proposed mediating model. Subsequently, PROCESS v2.16.3 (Model 4) examined the cooperative relationship among variables, calculating the mediating effect size and comparing specific indirect effects.

## Results

ANOVA identified significant effects of gender, English learning years and class satisfaction on scale scores to control in analysis. Peer support, self-efficacy, enjoyment and anxiety were significantly positively correlated (Table 2). Males scored lower in peer support and enjoyment than females (Table 3). English learning years impacted anxiety scores (Table 4). Class satisfaction affected peer support, self-efficacy and emotions (Table 5). No nationality differences emerged (Table 6).

**Table 2:** Inter-correlations and descriptive statistics.

	*M*	SD	1	2	3	4
1. PS	25.97	5.73	1			
2. GSE	28.86	5.28	0.57**	1		
3. FLE	41.79	8.14	0.70**	0.65**	1	
4.FLCA	24.96	6.93	0.59**	0.52**	0.54**	1

PS, peer support; GSE, general self-efficacy; FLE, foreign language enjoyment; FLCA, foreign language classroom anxiety. *M*, mean; SD, standard deviation. Asterisks indicate statistical significance: * *p* < 0.05, ** *p* < 0.01, *** *p* < 0.001.

**Table 3:** Results of t-test analysis for each scale on gender variables.

	Gender (*M*±SD)		*t*	*p*
	Male (*n*=221)	Female (*n*=260)		
1. PS	24.79±7.08	26.96±4.00	−4.04	0.000***
2. GSE	28.88±6.02	28.84±4.58	0.08	0.94
3. FLE	40.67±9.44	42.74±6.72	−2.74	0.007**
4. FLCA	24.86±7.88	25.05±6.01	−0.28	0.78

**Table 4:** One-way ANOVA results for each scale on years of learning English.

	Years of learning English (*M*±SD)			*F*	*p*
	1-6y (*n*=52)	7-10y (*n*=211)	10-13y(*n*=218) 10-1310-13y (*n*=218)		
PS	24.88±6.54	25.94±5.62	26.25±5.62	1.19	0.30
GSE	28.77±5.27	28.82±5.24	28.91±5.36	0.02	0.98
FLE	40.81±8.63	41.77±8.05	42.04±8.13	0.48	0.62
FLCA	24.60±6.89	24.00±6.60	25.99±7.13	4.58	0.01*

**Table 5:** One-way ANOVA results for each scale on satisfaction with the online English class.

	Satisfaction with online English class (*M*±SD)			*F*	*p*
	Dissatisfied (*n*=6)	Ok (*n*=125)	Very satisfied (*n*=350)		
PS	18.33±10.80	23.52±5.39	26.97±5.38	24.23	0.00**
GSE	24.17±12.40	26.69±4.64	29.71±5.08	18.74	0.00***
FLE	29.33±17.28	35.82±7.19	44.13±6.90	71.14	0.00***
FLCA	16.50±7.94	22.75±5.74	25.90±7.04	14.85	0.00***

**Table 6:** Results of t-test analysis for each scale on nationality variables.

	Nationality (*M*±SD)		*t*	*p*
	Han (*n*=433)	Minority (*n*=48)		
P S	25.99±5.75	25.77±5.60	0.247	0.805
GSE	28.96±5.32	27.94±4.97	1.270	0.205
FLE	41.83±8.16	41.38±8.09	0.370	0.712
FLCA	25.02±6.98	24.46±6.43	0.531	0.595

Table 2 presents the inter-correlations and descriptive statistics. Positive and significant associations were observed between peer support, self-efficacy, foreign language enjoyment, and anxiety.

Table 3 displays the descriptive statistics for gender variables. Independent samples *t*-tests were conducted to compare the scores of the four scales among male and female students. Results indicated a significant difference in the scores of male and female students on the peer support scale (*t = −4.04, p < 0.001*), with male students scoring considerably lower than female students. Additionally, male students rated significantly lower on the FLE scale than female students (*t = −2.74, p < 0.01*). However, no significant differences were found between male and female students in the GSE and FLCA scores.

Table 4 displays the results of descriptive statistics on years of English learning, followed by a one-way ANOVA to compare differences in scores for foreign language enjoyment and anxiety, peer support, and self-efficacy. The analysis reveals that anxiety scores significantly differed across the years of English learning (*F = 4.58, p < 0.05*), with lower scores for 7–10 years of study compared to 10–13 years of study (*p < 0.01*). PS, GSES, and FLE scores did not vary significantly across years of learning English.

Table 5 illustrates the descriptive statistics for each scale score on class satisfaction. A one-way ANOVA assessed disparities in foreign language enjoyment, English classroom anxiety, self-efficacy, and peer support scores. The peer support scale significantly differed in the student class satisfaction variable (*F = 24.23, p < 0.001*). The results indicated that students dissatisfied with their classes had lower scores than those with average, satisfied, and highly satisfied classes.

Similarly, the self-efficacy scale also demonstrated a significant difference (*F = 18.74, p < 0.001*), wherein students who were dissatisfied with their class had lower scores than those who were generally satisfied. Moreover, the PS scale revealed a significant difference (*F = 71.14, p < 0.001*), indicating lower scores on foreign language enjoyment and class satisfaction for students who were dissatisfied with their class than those who were generally satisfied and satisfied. Lastly, the anxiety scale exhibited a significant difference (*F = 14.85, p < 0.001*), demonstrating that students who were dissatisfied with their class had lower anxiety scores than generally satisfied and satisfied students. Table 6 shows no significant differences between the scores of nationality variables on the four scales.

### The mediating role of self-efficacy

Regression analyses controlling demographics supported self-efficacy partially mediating peer support’s effect on enjoyment and anxiety (Tables 7, 8). Peer support predicted higher enjoyment and anxiety, with self-efficacy reducing those relationships. Indirect effects indicated partial mediation. Findings suggest peer support may improve foreign language learning partially through enhancing self-efficacy.

**Table 7:** The mediating role of self-efficacy in peer support and foreign language enjoyment.

	FLE		GSE		FLE	
	*B*	*t*	*B*	*t*	*B*	*t*
Gender	0.30	0.60	−1.10**	−2.73	0.89	1.91
Nationality	0.60	0.72	−0.55	−0.83	0.89	1.18
Years of learning English	−0.36	−0.97	−0.20	−0.68	−0.25	−0.75
Satisfaction with Classes	5.12**	9.39	1.07*	2.47	4.55**	9.15
PS	0.86**	18.73	0.52**	14.19	0.59**	11.76
GSE					0.53**	10.16
*R* ^2^	0.49		0.33		0.58	
*F*	454.66 (1, 479)***		231.93 (1, 479)***		330.76 (1,478)***	

**Table 8:** The mediating role of self-efficacy in peer support and foreign language anxiety.

	FLCA	GSE	FLCA			
	*B*	*t*	*B*	*t*	*B*	*t*
Gender	−1.15*	−2.06	−1.10**	−2.73	−0.8	−1.26
Nationality	−0.17	−0.19	−0.55	−0.83	0.06	0.07
Years of learning English	0.81*	1.967	−0.20	−0.68	0.90*	2.29
Satisfaction with Classes	1.18	1.96	1.07*	2.47	0.72	1.25
PS	0.59**	11.52	0.52**	14.19	0.36**	6.25
GSE					0.43**	7.15
*R* ^2^	0.270	0.35	0.34			
*F*	34.87 (5,475)***	51.05 (5,475)***	40.64 (6,474)***			

Table 7 reports the relationship between peer support, self-efficacy, and foreign language enjoyment. Controlling for gender, nationality, years of English learning, and class satisfaction, Model 4 through SPSS software was used. The independent variable was PS score, while the dependent variable was enjoyment. Self-efficacy acted as the mediator. Likewise, Table 8 examines the association between peer support, self-efficacy, and foreign language anxiety. Model 4 through SPSS software was employed while controlling for gender, nationality, years of English learning, and class satisfaction. The independent variable was PS score, while the dependent variable was anxiety. Self-efficacy served as the mediator. Especially Table 7 revealed that peer support had a significant positive effect on both foreign language enjoyment (*β = 0.86, p < 0. 01*) and self-efficacy (*β = 0.52, p < 0. 01*). Furthermore, after including self-efficacy in the regression equation, peer support remained significantly associated with foreign language enjoyment (*β = 0.59, p < 0. 01*). The mediation effect ab = 0.28, Boot SE = 0.03, and 95% confidence interval was [0.15, 0.25], indicating that self-efficacy partially mediates the relationship between peer support and foreign language enjoyment.

Table 8 indicates that peer support has a significant positive effect on both anxiety (*β = 0.59, p < 0.01*) and self-efficacy (*β = 0.52, p < 0.01*). Furthermore, even after controlling for self-efficacy, peer support positively affected anxiety (*β = 0.36, p < 0.01*). These findings suggest that self-efficacy partially mediates the relationship between peer support and anxiety. The mediation effect ab = 0.23, Boot SE = 0.03, and the 95% confidence interval was [0.12, 0.25], indicating that self-efficacy partially mediates the relationship between peer support and foreign language anxiety.

### Qualitative findings

The semi-structured interviews with ten students from the online foreign language classes surveyed in the quantitative study covered four key areas: the influence of peer support on self-efficacy, the relationship between peer support-influenced self-efficacy and foreign language emotions, contextual factors moderating peer support, and recommendations for facilitating peer support. These areas provided a deeper understanding of students’ perspectives and experiences related to the three research questions, yielding converging evidence that informs practical recommendations for improving online language teaching and learning.

1. The influence of peer support on self-efficacy.

When asked how collaborating with peers impacts their self-efficacy, 70% of students described enhanced confidence from peer support. Informational support through exchanging language learning tips was especially beneficial for building grammar and vocabulary knowledge. Zhang shared: “Discussing grammar strategies with classmates helped me grasp difficult concepts better. My confidence for learning grammar increased”.

Emotional support like encouragement from peers also boosted self-efficacy. Li explained: “My study group kept me motivated to practice spoken English and gave me feedback on improving my pronunciation. Working together on our presentation really built my speaking confidence”.

However, some students revealed excessive peer exposure can provoke anxiety and diminish self-efficacy. Wang admitted: “Having all my classmates hear me speak makes me super nervous about mistakes, lowering my speaking confidence compared to smaller group discussions.” This highlights an optimal level of peer interaction is needed to enhance self-efficacy.

2. The relationship between peer support-influenced self-efficacy and foreign language emotions.

The interviews confirm the quantitative evidence that students’ self-efficacy strongly influences their foreign language emotions. Those with higher confidence reported increased enjoyment and motivation across language areas, while those with lower self-efficacy experienced more anxiety.

Chen described how improved grammar self-efficacy enhanced his motivation: “When my peers helped me understand grammar rules, I felt much more self-assured. Grammar lessons used to stress me out, but now I enjoy them and look forward to practicing”.

In contrast, Huang explained how lacking pronunciation confidence causes anxiety: “I do not feel very confident in my pronunciation compared to native speakers. I get really nervous that I’ll stumble over words in conversations”.

An important gender difference emerged female students reported more anxiety about mistakes and peer evaluation compared to males. Liu shared: “I feel more judged by the other girls when I make errors speaking English. I get more anxious about sounding awkward in front of them.” This aligns with the quantitative finding of a stronger self-efficacy-emotions relationship among females. In summary, higher self-efficacy strengthens positive emotions, while lower self-efficacy contributes to detrimental anxiety, with nuances across competencies and genders.

3. Contextual factors moderating peer support.

Students emphasized factors like large class sizes and minimal teacher involvement reduced benefits of online peer support. With small classes and strong teacher presence, peer support effects were amplified. Jin said, “In large online classes, it’s hard to build personal connections. Other students are just names on the screen.” Dong shared, “Interacting through discussion boards feels anonymous and impersonal. It’s not the same as face-to-face interaction”.

However, in smaller classes, online peer support was valuable. Li stated: “Having around 15 students in breakout rooms allowed us to get to know each other and feel comfortable asking for help. We formed study groups that extended beyond the classroom”.

Strong faculty presence also helped maximize peer support online. Huang explained: “Our professor actively participated in discussions and group work. This created a supportive environment where we knew we could rely on each other for feedback and questions”.

These insights indicate contextual factors like class size and faculty involvement moderate the benefits students obtain from online peer support, impacting their self-efficacy, emotions, and learning.

4. Recommendations for facilitating peer support.

Students recommended smaller online breakout groups, regular collaborative projects, teacher-guided community-building activities, and constructive feedback guidance to optimize peer support. Zhang summarized: “Having the teacher create more interaction chances in smaller teams would really build my confidence through exchanging tips with peers”.

An interesting gender difference emerged, with female participants mentioning peer evaluation anxiety more often. For example, Liu, a female student, explained: “I get really worried about making pronunciation mistakes when I have to do oral presentations in front of the whole class. The other girls seem to have better accents and vocabulary than me. I feel like they are judging me whenever I stutter or mispronounce something”.

Another female interviewee, Zhang, shared: “I used to feel excited about participating in online discussions. But then I noticed some female classmates making critical comments about others’ writing mistakes. Now I feel a lot more hesitant to post in the forums because I do not want them negatively evaluating my language abilities”.

This suggests higher performance pressures for female language learners in online contexts. While self-efficacy can increase motivation, excessive confidence may also breed unnecessary stress. A balanced view recognizing one’s strengths and weaknesses may optimize learning emotions.

In summary, the interviews provided converging evidence that peer support enhances self-efficacy and learning emotions, while uncovering unexpected moderating factors like overexposure anxiety and gender differences. Students’ insights complement the quantitative data to give a holistic understanding that informs practical recommendations for improving online language teaching and learning environments sensitive to gender needs and optimal peer collaboration.

The mixed methods approach combining open-ended interviews with closed-ended surveys yielded a multifaceted investigation of the complex relationships between peer support, self-efficacy, emotions, gender factors, contextual influences, and ideal online pedagogy.

## Discussion

### Integration of quantitative and qualitative findings

This mixed methods study explored the interplay between peer support, foreign language emotions, and self-efficacy in online language learning contexts, yielding valuable insights into how these factors impact students’ experiences.

This quantitative research examined the relationships among peer support, self-efficacy, enjoyment, and anxiety in a sample of 481 Chinese undergraduates enrolled in an online English course. The study employed demographic controls to ensure the validity of the results. The findings revealed that peer support had a significant positive effect on both enjoyment and anxiety levels. Furthermore, self-efficacy was found to partially mediate the relationship between peer support and enjoyment/anxiety, with peer support strengthening self-efficacy, which, in turn, enhanced enjoyment and anxiety. These results suggest the critical mediating role of self-efficacy in the relationship between peer support and emotional experiences in foreign language learning. The study also revealed that male students reported lower peer support and enjoyment than their female counterparts. Moreover, more years of learning English were associated with higher anxiety levels. Dissatisfaction with the course was linked to decreased peer support, self-efficacy, enjoyment, and anxiety. However, no significant differences emerged based on nationality. These findings underscore the interconnected roles of peer support, self-efficacy, and emotions in online foreign language learning.

The quantitative findings indicate that peer support in digital foreign language classes can bolster students’ enjoyment and confidence while also exacerbating anxiety. This aligns with prior research demonstrating peer interaction has mixed implications for foreign language learning (Chen et al., 2022; Zheng and Zhou, 2023). Self-efficacy appears to serve as a mechanism through which peer support influences affective experiences, supporting previous research (Mastrocola and Flynn, 2017).

Additionally, gender, prior language experience and course satisfaction moderate the effects of peer support. Instructors should facilitate collaborative activities with awareness of individual differences.

The in-depth interviews provided valuable insights that expand upon the quantitative results regarding peer support, self-efficacy, emotions, and optimal online language learning environments. The qualitative data converged with and complemented the survey findings in multiple ways.

First, students’ descriptions of how peer support enhanced their self-efficacy align with the quantitative relationship found between peer support and self-efficacy. The interviews shed light on the mechanisms, such as exchanging learning tips and encouragement, that may drive this relationship. However, they also uncovered potential limiting factors, such as overexposure anxiety, that can diminish self-efficacy. This underscores the need for an optimal level of peer interaction.

Second, students vividly illustrated how self-efficacy influences emotions, for example describing how improved grammar confidence increased motivation and enjoyment. Their accounts converge with the quantitative evidence of strong links between self-efficacy and emotions. Importantly, the interviews revealed nuances, such as gender differences in evaluation anxiety, that were not captured in the quantitative data.

Third, the interviews highlighted contextual moderators, such as class size and teacher involvement, that may impact peer support benefits. Students felt that small classes and strong teacher presence optimized online peer support, aligning with the quantitative moderator analysis. Their perspectives provide concrete recommendations for facilitation.

Finally, the qualitative findings converged with but also expanded upon the survey results to provide a comprehensive understanding of the complex interrelationships between the key variables. The mixed methods approach was beneficial for obtaining multifaceted insights into optimal online language learning environments sensitive to peer support needs and gender differences.

Overall, the integration of quantitative and qualitative findings provides a comprehensive understanding of the complex interplay between peer support, self-efficacy, emotions, and gender factors in online language learning contexts, yielding valuable insights into how these factors impact students’ experiences.

### Peer support and foreign language emotions: the mediated role of self-efficacy

This mixed methods investigation provides empirical evidence that online peer support significantly predicts self-efficacy beliefs and key emotions, namely enjoyment and anxiety, in the context of language learning. Qualitative findings further shed light on the mechanisms underlying these relationships.

Drawing upon Vygotsky’s sociocultural theory, this study demonstrates that collaborating with peers can provide cognitive and emotional scaffolding that enhances self-efficacy and enjoyment (Vygotsky, 1978). However, over-reliance on peer support could hinder the development of autonomous mastery. The control-value theory also elucidates the social shaping of emotions, highlighting how peer interactions can enhance perceived control and convey task value, while unfavorable comparisons can undermine control beliefs and dampen value appraisals (Pekrun, 2006).

Moreover, the online context introduces unique dynamics whereby digital tools enable convenient collaboration, but the physical absence exacerbates feelings of isolation. Balancing meaningful social connections with developing self-regulatory skills in virtual settings is crucial.

Quantitative results indicate that self-efficacy partially mediates the link between peer support and emotions, consistent with social cognitive theory’s tenets on the pivotal role of self-efficacy in informing cognitive appraisals and affective responses. Qualitative findings suggest that collaborating with peers provides enactive mastery experiences and vicarious learning opportunities that strengthen self-efficacy. However, excessive praise may provoke anxiety by raising standards, underscoring the importance of maintaining a balanced and growth-oriented mindset.

While this study significantly advances our understanding of peer influence processes, further research is necessary to track bidirectional and temporal effects. Educators should leverage social resources to enhance self-efficacy and enjoyment while mitigating the risks of over-dependence and isolation in digital spaces.

### Practical implications for online learning

This study provides valuable practical implications for optimizing the design of online foreign language courses to support learner motivation and emotional well-being.

First, varied collaborative activities should be embedded to provide peer support, enabling modeling, co-regulation, and motivational reinforcement. However, over-reliance on peers should be avoided by incorporating independent practice and self-reflection.

Second, instructors should cultivate a socially supportive climate minimizing negative peer evaluation. Shared guidelines and etiquette norms can maintain respectful interactions. Praise should reinforce effort over fixed abilities.

Third, self-efficacy can be nurtured through structured goal setting, strategy instruction, and reflective journaling on progress. However, growth mindset principles should be emphasized over unrealistic self-appraisals.

Fourth, individual mentoring and small group discussions could support students struggling with isolation or evaluation fears. Virtual coffee chats can also foster casual connections.

Finally, varied modalities like student videos, chat, and live conferencing should maximize social cues. Synchronous gatherings and informal interactions can mitigate remote learning challenges.

This research provides practical guidelines for leveraging online peer support and self-efficacy-enhancing practices to optimize foreign language learning experiences. However, contextual and individual differences must be considered in implementation.

### Limitations and future research

Although this study provides valuable insights, it has several limitations that suggest directions for future research. Firstly, the sample only comprised Chinese university students in one online English course. Therefore, additional studies should examine whether the findings can be generalized to other cultural settings, age groups, proficiency levels, and language domains.

Secondly, this study relied on self-report measures. To provide more objective assessments, future research could incorporate behavioral observations, physiological indicators, and peer ratings. Additionally, think-aloud protocols could be used to reveal cognitive-affective processes.

Thirdly, this study only examined emotional and informational peer support. Therefore, future research should investigate the effects of appraisal support, such as performance feedback. Furthermore, the effects of opposing or competitive peer interactions also warrant attention.

Finally, additional mediators and moderators could be incorporated to extend the proposed model, such as classroom goal structures, teacher support, perfectionism, or gender role beliefs. Additionally, exploring whether the findings can be generalized to face-to-face contexts would be informative.

Despite these limitations, this study significantly contributes to the literature by using an integrated mixed methods approach to empirically demonstrate the interconnected relationships between online peer support, self-efficacy, and foreign language learning emotions. Future research can build on these findings to deepen our understanding of the socio-emotional experiences of students in digitally mediated learning environments.

## Conclusion

This study found that peer support positively predicted self-efficacy, enjoyment, and anxiety, with self-efficacy partially mediating the effects. These results demonstrate the roles of social and psychological factors in online language learning. The practical implications point to strategies for optimizing peer interactions and self-efficacy development. However, the study has limitations in the sample and measures. Future studies should examine additional factors and contexts. Overall, the findings highlight the importance of social and motivational support in digital education.

## Data availability statement

The original contributions presented in the study are included in the article/supplementary material, further inquiries can be directed to the corresponding author.

## Ethics statement

The requirement of ethical approval was waived by research institution for the studies involving humans because research institution. The studies were conducted in accordance with the local legislation and institutional requirements. The participants provided their written informed consent to participate in this study. Written informed consent was obtained from the individual(s) for the publication of any potentially identifiable images or data included in this article.

## Author contributions

The author confirms being the sole contributor of this work and has approved it for publication.
